# Automated Detection of Reduced Ejection Fraction Using an ECG-Enabled Digital Stethoscope: A Large Cohort Validation

**DOI:** 10.1016/j.jacadv.2025.101619

**Published:** 2025-02-20

**Authors:** Ling Guo, Gregg S. Pressman, Spencer N. Kieu, Scott B. Marrus, George Mathew, John Prince, Emileigh Lastowski, Rosalie V. McDonough, Caroline Currie, John N. Maidens, Hussein Al-Sudani, Evan Friend, Deepak Padmanabhan, Preetham Kumar, Edward Kersh, Subramaniam Venkatraman, Salima Qamruddin

**Affiliations:** a Eko Health, Inc, Emeryville, California, USA; b Jefferson Einstein Hospital, Thomas Jefferson University, Philadelphia, Pennsylvania, USA; c Prairie Education & Research Cooperative, Springfield, Illinois, USA; d Sri Jayadeva Institute of Cardiovascular Sciences and Research, Bengaluru, India; e Heart and Vascular Institute, MedStar Washington Hospital Center, Washington, District of Columbia, USA; f Sutter Care at Home, Fairfield, California, USA; g Ochnser Heart and Vascular Institute, Ochsner Medical Center, New Orleans, Louisiana, USA

**Keywords:** artificial intelligence, asymptomatic left ventricular systolic dysfunction, digital health, heart failure detection, reduced ejection fraction

## Abstract

**Background:**

Asymptomatic left ventricular systolic dysfunction (ALVSD) affects 7 million globally, leading to delayed diagnosis and treatment, high mortality, and substantial downstream health care costs. Current detection methods for ALVSD are inadequate, necessitating the development of improved diagnostic tools. Recently, electrocardiogram-based algorithms have shown promise in detecting ALVSD.

**Objectives:**

The authors developed and validated a convolutional neural network (CNN) model using single-lead electrocardiogram and phonocardiogram inputs captured by a digital stethoscope to assess its utility in detecting individuals with actionably low ejection fractions (EF) in a large cohort of patients.

**Methods:**

2,960 adults undergoing echocardiography from 4 U.S. health care networks were enrolled in this multicenter observational study. Patient data were captured using a digital stethoscope, and echocardiograms were performed within 1 week of data collection. The algorithm's performance was compared against echocardiographic EF (EF measurements, categorizing EF as normal and mildly reduced [>40%] or moderate and severely reduced [≤40%]).

**Results:**

The CNN model demonstrated an area under the receiver operating characteristic curve of 0.85, with a sensitivity of 77.5%, specificity of 78.3%, positive predictive value of 20.3%, and negative predictive value of 98.0%. Among those with an abnormal artificial intelligence screen but EF >40% (false positives), 25% had an EF between 41%-49% and 63% had conduction/rhythm abnormalities. Subgroup analyses indicated consistent performance across various demographics and comorbidities.

**Conclusions:**

The CNN model, utilizing a digital stethoscope, offers a noninvasive and scalable method for early detection of individuals with EF ≤40%. This technology has the potential to facilitate early diagnosis and treatment of heart failure, thereby improving patient outcomes.

Heart failure remains a significant global health concern. Congestive heart failure affects 5 million people and consumes more than 30 billion in health care expenditure.^1^^,^^2^ Asymptomatic left ventricular systolic dysfunction(LVSD) has an estimated prevalence of 3% to 6%, affecting approximately 7 million people, and is at least as common in the community as systolic heart failure.^3^ Because it often occurs in the absence of known cardiovascular disease, this condition may go unrecognized and untreated.^3^ Asymptomatic LVSD, classified as stage B heart failure, is defined as structural heart disease without current or prior symptoms. Early intervention is known to be effective, yet late diagnosis persists, leading to heart failure being identified in acute care settings despite earlier documentation of potential symptoms in primary care.^4^ Delayed diagnosis contributes to the unacceptably high mortality rates associated with heart failure.^5^ Additionally, the underutilization of echocardiography and late diagnosis of heart failure incur substantial costs for health care systems.^6^ Underlying this is the lack of simple and scalable screening tools available for early detection and intervention.^7^

Since its invention over 200 years ago, the stethoscope has remained an indispensable tool in the clinical examination of patients.^8^ With advancements in technology, notably the advent of artificial intelligence (AI) and digital stethoscopes capable of capturing both heart sound (phonocardiogram [PCG]) and electrocardiogram (ECG) signals, diagnostic capabilities of the stethoscope have substantially improved for a wide range of users. While echocardiography remains the gold standard for diagnosing low ejection fraction (EF), its widespread use is limited by resource constraints, requirement for extensive training, operator-dependent variability, and cost. PCG and ECG signals, captured by a digital stethoscope and processed through AI-enabled algorithms, provide a noninvasive method to detect a low EF that can be used equally effectively by individuals with all levels of training and experience.^1^^,^^9^

Digital stethoscopes have already demonstrated their ability to improve diagnostic accuracy in several clinical contexts, including the detection of heart murmurs and lung sounds. By incorporating AI into these devices, we aim to further enhance their utility in the detection of low EF, offering a noninvasive, accessible tool for early heart failure diagnosis.^10^^,^^11^

To our knowledge, few studies have explored AI-enhanced digital stethoscopes for detecting low EF. These either did not incorporate PCG data into their algorithm or involved different patient populations (eg, peripartum individuals). The usage of PCG for the detection of low EF is new in this study. Models based on PCG signal alone are less predictive for low EF compared to models based on ECG alone. In a previous study,^12^ it was observed that a model using PCG alone had an area under the curve of 77.8%, while a model using single-lead ECG alone had an area under the curve of 80.4% for detecting low EF. In the current study, we show that a model that simultaneously analyzes synchronously captured PCG and ECG performs better than a model that uses single-lead ECG alone. Our study incorporates both single-lead ECG and PCG signals into the algorithm to detect LVEF ≤40% in a diverse, real-world cohort. Our aim was to validate the predictive accuracy of this algorithm in a real-world setting in identifying patients with an EF of 40% or less, thus facilitating early detection and management.

## Methods

### Study population and setting

This multicenter observational study was conducted using data from 4 geographically distinct U.S. health care networks: Jefferson Einstein Philadelphia Hospital (Pennsylvania), Prairie Cardiovascular Consultants (Illinois), MedStar Health Research Institute (Maryland), and Ochsner Heart and Vascular Institute (Louisiana). The study population consisted of adults aged 18 years and older undergoing a clinically indicated echocardiogram for any reason within 7 days of study procedures and who were willing and capable of providing informed consent. Nonprobability, exhaustive sampling method was used to enroll individuals in the study. Exclusion criteria included individuals who were unwilling or unable to provide informed consent, as well as hospitalized patients. Prior diagnosis of heart failure or reduced EF did not preclude patients from participating in the study. Patient demographic data, including age, race/ethnicity, sex, and medical history, were collected. The responsible Institutional Review Boards and ethics committees of the participating centers approved this study.

### Algorithm development

The development of the low EF convolutional neural network (CNN) model built upon previous work conducted by Attia et al.^1^^,^^9^ Briefly, the model uses an ensemble of 3 CNNs. The primary component of the ensemble (referred to as the ECG model) was trained on 44,959 12-lead ECG and echocardiogram pairings from the Mayo Clinic database.^1^ The inputs to the ECG model were single leads from the 12-lead ECGs in the Mayo Clinic data set and the model was trained to predict whether LVEF was normal (>40%) or low (≤40%). Information on which ECG lead was being used was not provided to the ECG model. The model was trained in this manner so that the resulting model is robust and not sensitive to the location/orientation at which the ECG lead is captured. To further refine the algorithm, 18,999 paired PCG and ECG recordings from 1,852 subjects sourced from a proprietary data set, referred to as the Eko Training Dataset, were utilized. The training data set was acquired from 8 clinical study sites (both within and outside the United States) independent of the study sites used for testing. Subjects in the training data set underwent transthoracic echocardiography within 30 days of the ECG-PCG recordings to ensure accurate ground truth labeling. These data enabled the fine-tuning of the ECG model and the creation of 2 additional models in the ensemble that utilize PCG as well, enabling the low EF CNN model to accept both single-lead ECG and PCG inputs.

The final model is an ensemble of 3 models (ECG model, PCG model, and PCG-ECG model). The input to the PCG model is the one-dimensional time-series or PCG waveform at 2,000 Hz, and the input to the PCG-ECG model is synchronously captured PCG and ECG waveforms. All 3 models have a ResNet^13^ style architecture and they all have 2 nodes in the output layer, one representing the probability of “low ejection fraction,” and one representing the probability of “normal ejection fraction” (which is 1 - probability of “low ejection fraction”). The probability outputs from the 3 models were averaged to get the final probability output of the ensemble model. A simple threshold was then applied on the final averaged probability output. If the model output for low EF is equal or greater than the threshold, the output of the Ejection Classification Algorithm is “low ejection fraction,” otherwise the output is “normal ejection fraction.” The probability threshold for the final ensemble model was determined using a separate internal validation set (which was not used for training the model or the final evaluation). The threshold was chosen to maximize the sum of sensitivity and specificity on the internal validation set.

The Eko Test Dataset includes paired ECG and PCG recordings and echocardiograms from 2,960 unique subjects. The data set was collected exclusively from the 4 study sites using the predefined inclusion and exclusion criteria and represents a wholly independent test set with no overlap with the training data. This test set was powered to estimate sensitivity and specificity with the lower bound of the 95% CI to be above 70%, assuming a sensitivity of 80% and a low EF prevalence of ∼15%. Recruitment continued until 210 subjects with low EF were included in the data set to ensure sufficient precision for sensitivity estimation.

### Study procedures

**Figure 1:**
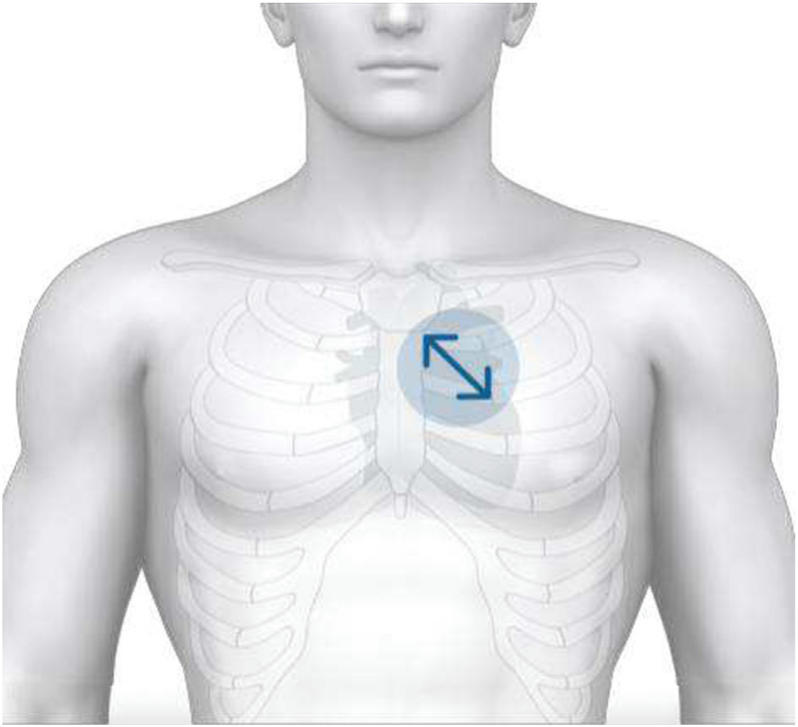
Placement of the Eko DUO Digital Stethoscope in a Modified Lead II Orientation The Eko DUO digital stethoscope is placed on the chest in a modified lead ii orientation, with the device positioned along the midline of the sternum, extending diagonally toward the left hip. This placement is designed to optimize the quality of both the ECG and phonocardiogram (PCG) signals for accurate ejection fraction analysis. ECG = electrocardiogram.

For data capture, a single 15-second ECG and PCG recording was obtained using the Eko DUO digital stethoscope (Eko Health, Inc). The stethoscope was placed in a modified lead II orientation (Figure 1) on patients in a seated position. Previous validation work determined that this position and orientation provided the best algorithm performance.^14^ All participants underwent echocardiography within a maximum of 7 days of the corresponding ECG and PCG recordings.

### EF determination

The EF of each person was measured by the echocardiogram machine's integrated cardiac quantification software at the time of the echocardiogram or a manual biplane Simpson’s measurement. For all included patients, EF measurements were then over-read by board-certified cardiologists at each site.^15^ The final EF determination was based on the EF provided in the clinical report and, for this study, was dichotomized as ≤40% or >40%. Interobserver variability of EF measurements was performed in a subset of 194 patients from 2 independent readers and was 0.706 (Cohen’s kappa).

### CNN model analysis

**Figure 2:**
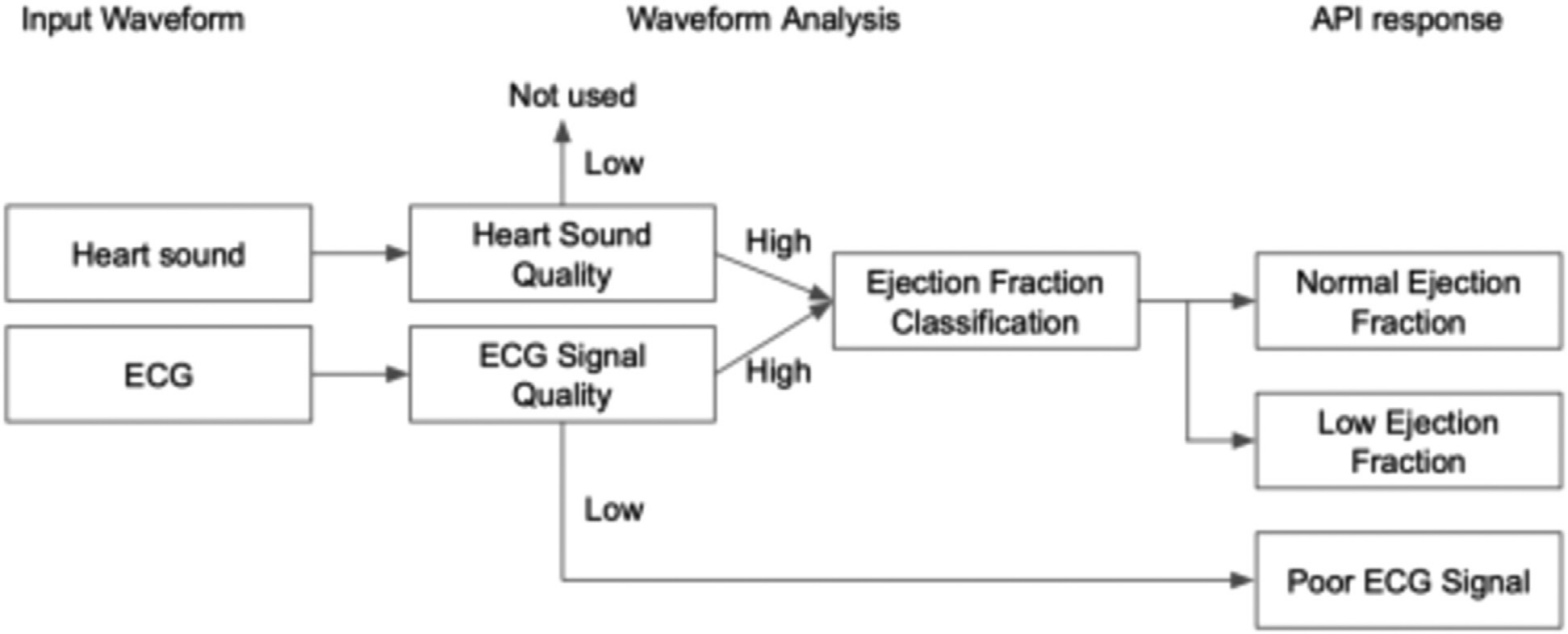
Schematic of Eko Low EF AI Operational System This schematic outlines the operational workflow of the Eko low EF AI system. Input waveforms from heart sound (PCG) and electrocardiogram (ECG) are analyzed for signal quality. If the ECG signal quality is sufficient, the data are processed for ejection fraction classification. The AI model then outputs one of 3 possible results: normal ejection fraction, low ejection fraction, or poor ECG signal, based on the quality of the data and the classification outcome. AI = artificial intelligence; API = Application Programming Interface; EF = ejection fraction; PCG = phonocardiogram.

The signal quality of the ECG and PCG recordings was assessed using a deep learning classifier previously validated against expert/cardiologist consensus, serving as the ground truth. Only recordings deemed to have good signal quality underwent EF classification. Specifically, if the ECG signal quality was assessed as poor, no EF classification result was provided, irrespective of the PCG signal quality. In cases where the ECG signal quality was deemed good, but the PCG signal quality was poor, only the ECG model was utilized. Conversely, when both the ECG and PCG signal qualities were satisfactory, an ensemble model was employed (Figure 2). The algorithm identified instances of “low ejection fraction detected” (defined as an EF of ≤40%) when the output of the EF classification algorithm surpassed a predetermined threshold. The potential AI outputs for low EF were “low ejection fraction detected,” “normal ejection fraction detected,” or “poor ECG signal.”

### Outcomes

The primary aim of this study was to determine the performance of an ECG-enabled digital stethoscope in identifying patients with reduced EF of ≤40% using an algorithm derived from single-lead ECG and PCG inputs. The primary endpoints included the performance metrics sensitivity and specificity. Secondary endpoints included positive predictive value (PPV), negative predictive value (NPV), and diagnostic yield (Central Illustration).

**Central Illustration:**
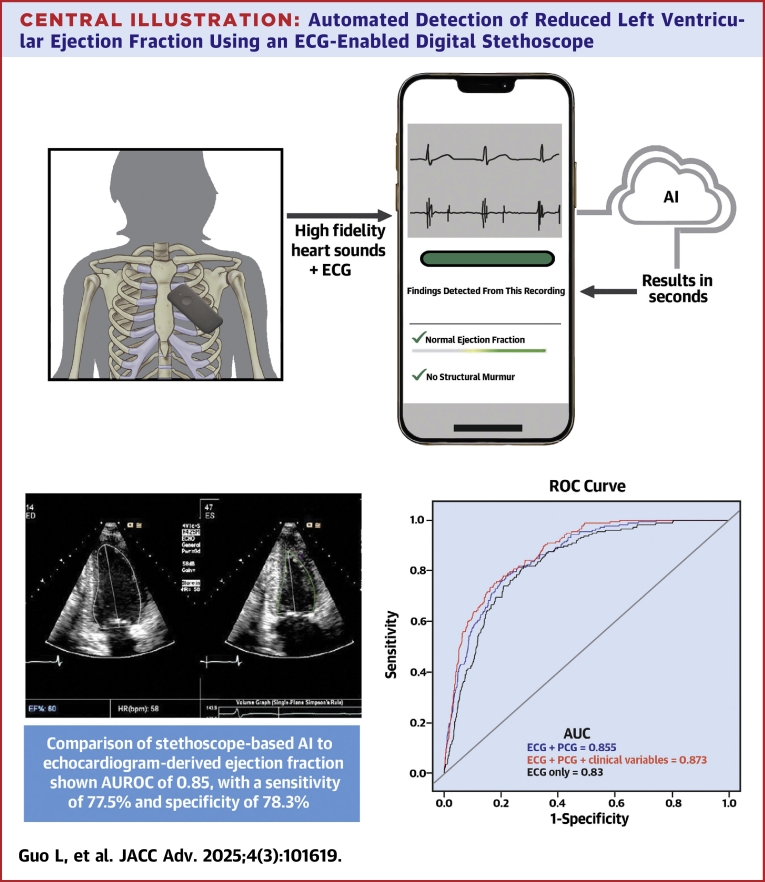
Automated Detection of Reduced Left Ventricular Ejection Fraction Using an ECG-Enabled Digital Stethoscope Workflow and outcomes of an AI-enabled digital stethoscope for early detection of asymptomatic left ventricular systolic dysfunction. The device captures single-lead ecg and heart sound data, which is rapidly analyzed via a cloud-based algorithm to identify patients with ejection fractions (EF) ≤40%. The convolutional neural network model showed an AUROC of 0.85, with 77.5% sensitivity and 78.3% specificity, demonstrating its potential as a scalable, noninvasive tool for early heart failure diagnosis. Abbreviations as in Figure 1, Figure 2, Figure 3.

### Statistical analysis

Patient demographic and clinical data for the entire cohort are presented using descriptive statistics. A comprehensive panel of algorithm performance metrics, including sensitivity, specificity, PPV, NPV, and diagnostic yield, were calculated. Diagnostic yield is defined as the percentage of recordings that the algorithm recognized as having good signal quality and which were suitable for a low EF interpretation. Exploratory subgroup analyses were conducted to assess the effects of participant characteristics such as sex, race, and medical history on algorithm performance.

Finally, as an exploratory analysis, we tested the performance of both an “augmented” model that included low EF AI output and pertinent clinical variables known to be associated with heart failure with reduced EF (ie, age, biological sex, body mass index, hypertension, diabetes, coronary artery disease, and presence of a pacemaker^16^, ^17^, ^18^, ^19^, ^20^, ^21^) and a “clinical” model, using the same clinical parameters, but without low EF AI output. Backward selection was employed to identify the most relevant variables for model inclusion. Model performance, as indicated by area under the receiver operating characteristic curve (AUROC), was compared to that of the “baseline” model (ie, univariable prediction of reduced EF ≤40% based on low EF AI output alone) using DeLong’s test. A *P* value of < 0.05 was considered statistically significant. Statistical analyses were performed using RStudio (2023.6.1.524).

## Results

### Demographic data

From October 2020 to May 2022, 2,988 patients (median age 66 years [IQR: 56-75], 51% female) were enrolled. Due to missing echocardiographic EF calculation, 28 subjects from the 4 sites were excluded from the study, resulting in a final cohort of 2,960. A total of 210 patients (7.1%) had an EF of ≤40%, as identified by their study echocardiogram. Detailed demographic information can be seen in Table 1. The breakdown by participating site can be seen in Supplemental Table 1. The majority of participants were referred through primary care (55.2%), followed by general cardiology (18.2%), emergency care (11.7%), other (13.6%), and urgent care (0.24%); for the remaining 1.0%, information was not available. The most commonly identified comorbidities were hypertension (2,296, 77.6%), obesity (1,525, 51.5%), coronary artery disease (1,053, 35.6%), and diabetes (800, 27.0%) (Table 1). The inclusion criteria were patients scheduled for an echocardiogram for any reason. Among this cohort, the most common symptomatic indication for the echocardiogram was dyspnea (1,248, 42%).

**Table 1:** Demographic and Medical Information of Enrolled Patients

	All (N = 2,960)	EF ≤40% (n = 210)	EF >40% (n = 2,750)	*P* Value^a^
Age, y	66 (56-75)	69 (59-77)	66 (55-74)	0.006
Female	1,508 (51)	70 (33.3)	1,439 (52.3)	<0.001
BMI, kg/m^2^	30.3 (26.0-35.2)	29 (25.5-33.5)	30.4 (26.0-35.2)	0.02
Ejection fraction, %	60 (53-86)	34 (30-37)	60.5 (55-65)	N/A
Race				0.05
White	2,011 (67.9)	127 (60.5)	1,884 (68.5)	
Black or African American	748 (25.3)	66 (31.4)	682 (24.8)	
American Indian or Alaska Native	28 (0.95)	0 (0)	28 (1.0)	
Asian	21 (0.71)	1 (0.5)	20 (0.7)	
Native Hawaiian or other Pacific Islander	5 (0.17)	1 (0.5)	4 (0.1)	
Other	145 (5.0)	15 (7.1)	132 (4.8)	
Prevalence of comorbidities^b^				
Hypertension	2,296 (77.6)	177 (84.3)	2,119 (77.1)	0.02
Coronary artery disease	1,053 (35.6)	114 (54.3)	939 (34.1)	<0.001
Diabetes mellitus	800 (27.0)	76 (36.2)	724 (26.3)	0.002
Cardiomyopathy^c^	489 (16.5)	116 (55.2)	373 (13.6)	<0.001
Obesity (BMI>30 kg/m^2^)	1,525 (51.5)	90 (42.9)	1,435 (52.2)	0.01
Permanent atrial fibrillation	74 (2.5)	12 (5.7)	62 (2.3)	0.004
Chronic obstructive pulmonary disease	289 (9.8)	29 (13.8)	260 (9.5)	0.05
Renal failure	258 (8.7)	29 (13.8)	260 (9.5)	0.12
Obstructive sleep apnea	429 (14.5)	26 (12.4)	403 (14.7)	0.42
Left bundle branch block	155 (5.2)	38 (18.1)	117 (4.3)	<0.001
Receiving cardiotoxic drugs	26 (0.88)	0 (0)	26 (0.95)	0.30
Aortic stenosis	157 (5.3)	10 (4.8)	147 (5.3)	0.84
Myocardial infarction	379 (12.8)	56 (26.7)	323 (11.7)	<0.001

Values are median (IQR) or n (%).

EF = ejection fraction.

a Chi-squared for continuous variables, Wilcoxon rank-sum for categorical variables.

b The total exceeds 100% because some subjects had more than one risk factor.

c Cardiomyopathy refers to diagnosis of any disease of the heart muscle, including heart failure.

### Performance of low EF AI

**Figure 3:**
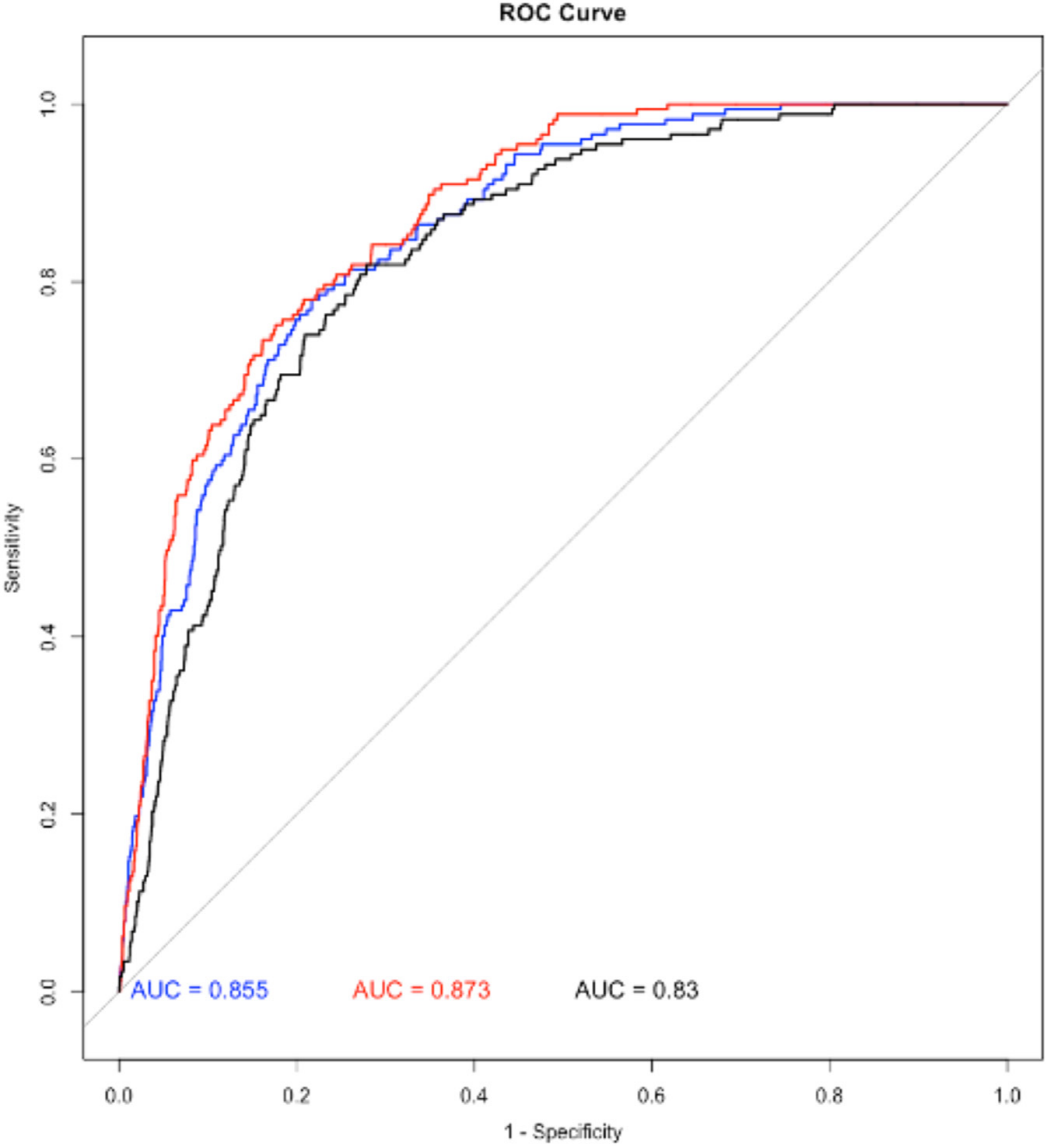
ROC Curve for the Entire Cohort From 4 U.S. Sites The graph above shows, in blue, the ROC curve and associated AUC for reduced EF detection by the convolutional neural network (CNN) model alone (ECG + PCG model). In red are the results when clinical variables (age, sex, BMI, and presence of pacemaker) are added to the algorithm output in a multivariable model. In black, we show the results of the ECG-only model (ie, without PCG input). Removing the AI output from that model resulted in a significant reduction in AUROC to 0.710 (*P* < 0.001). All subsequent results shown in this publication are based on the ECG-PCG low EF AI unadjusted model. AUC = area under the curve; BMI = body mass index; AUROC = area under the receiver operating characteristic curve; other abbreviations as in Figures 1 and 2.

The overall performance of the EF classification algorithm resulted in an AUROC of 0.852 (95% CI: 0.826-0.878), sensitivity of 77.5% (95% CI: 70.7-83.4), specificity of 78.3% (95% CI: 76.7-79.9), PPV of 20.3% (95% CI: 17.3-23.5), NPV of 98.0% (95% CI: 97.3-98.6), and a diagnostic yield of 90.7% (95% CI: 89.6-91.7). An augmented logistic regression model including age, sex, presence of a pacemaker, and body mass index in addition to low EF AI output increased the AUROC slightly yet significantly from 0.85 to 0.87 (*P* < 0.001). The presence of clinical variables alone (ie, without the AI output included in the model) resulted in a significant reduction in AUROC to 0.710 (*P* < 0.001) (Figure 3, Table 2).

**Table 2:** Performance Metrics of Low EF AI Classification Against Ground Truth of Echocardiography

	Sensitivity	Specificity	PPV	NPV	Diagnostic Yield
Low EF AI	77.5 (70.7-83.4)	78.3 (76.7-79.9)	20.3 (17.3-23.5)	98.0 (97.3-98.6)	90.7 (89.6-91.7)

Values are % (95% CI).

AI = artificial intelligence; NPV = negative predictive value; PPV = positive predictive value; other abbreviation as in Table 1.

### Exploratory subgroup analyses

#### By patient characteristic

A subanalysis demonstrating the performance of the low EF AI for subjects with specific cardiovascular conduction disorders is provided in Table 3. These patients showed a decreased specificity compared to other patients. No significant differences were observed, although the subgroups were small with wide confidence intervals. Further subgroup analyses were conducted to assess performance as stratified by age, biological sex, and race (Table 4). Specificity varied slightly across biological sex (male: 73.6% [95% CI: 71.3-75.8] vs female: 81.8% [95% CI: 79.7-83.9]) and was lower for patients aged >70 years (70.7% [95% CI: 67.7-73.6]) vs the entire cohort (78.3% [95% CI: 76.7-79.9]). No other significant differences were observed.

**Table 3:** Performance Metrics in Patients With Specific Cardiac Conduction Disorders

	Recordings	Sensitivity	Specificity	PPV	NPV	Diagnostic Yield
Permanent atrial fibrillation	74	100.0 (71.5-NA)	50.0 (35.8-64.2)	29.7 (15.9-47.0)	100.0 (86.8-NA)	85.1 (75.0-92.3)
Left bundle branch block	155	93.9 (79.8-99.3)	28.2 (19.7-37.9)	29.5 (21.0-39.2)	93.5 (78.6-99.2)	87.7 (81.5-92.5)
Pacemaker	278	87.3 (76.5-94.4)	41.1 (33.8-48.8)	34.8 (27.4-42.8)	90.0 (81.2-95.6)	85.6 (80.9-89.5)

Values are n or % (95% CI).

Abbreviations as in Table 2.

**Table 4:** Performance Metrics Across Exploratory Subgroup Analyses

	Recordings	Sensitivity	Specificity	PPV	NPV	Diagnostic Yield
Biological sex						
Male	1,451	79.5 (71.0-86.4)	74.4 (71.8-76.8)	23.5 (19.4-28.0)	97.3 (96.1-98.3)	89.5 (87.8-91.1)
Female	1,509	73.8 (60.9-84.2)	81.9 (79.7-83.9)	15.8 (11.8-20.6)	98.5 (97.6-99.2)	91.8 (90.3-93.1)
Age (y)						
18-30	94	100 (15.8- nan)	87.1 (78.0-93.4)	15.4 (1.9-45.4)	100 (95.1-nan)	92.6 (85.3-97.0)
31-50	400	68.8 (41.3-89.0)	87.0 (83.1-90.4)	19.3 (10.0-31.9)	98.4 (96.3-99.5)	92.7 (89.8-95.1)
51-70	1,274	73.6 (61.9-83.3)	81.6 (79.2-83.9)	20.9 (16.0-26.4)	97.9 (96.8-98.7)	91.4 (89.8-92.9)
>70	1,192	81.8 (72.2-89.2)	70.7 (67.7-73.6)	20.2 (16.1-24.7)	97.7 (96.3-98.7)	89.0 (87.1-90.7)
Race						
Black/African American	748	72.2 (58.4-83.5)	78.6 (75.2-81.8)	22.7 (16.6-29.7)	97.0 (95.1-98.3)	90.4 (88.0-92.4)
White	2,011	81.7 (73.1-88.4)	77.4 (75.4-79.4)	18.6 (15.2-22.4)	98.5 (97.7-99.1)	91.0 (89.7-92.3)
Other	147	64.3 (35.1-87.2)	85.3 (77.6-91.2)	34.6 (17.2-55.7)	95.2 (89.1-98.4)	88.4 (82.1-93.1)
Asian	21	100.0 (2.5-NA)	88.2 (63.6-98.5)	33.3 (0.8-90.6)	100.0 (78.2-NA)	85.7 (63.7-97.0)
American Indian or Alaska Native	28	-	92.3 (74.9-99.1)	0.0 (NA-84.2)	100.0 (85.8-NA)	92.9 (76.5-99.1)

Values are n or % (95% CI).

Abbreviations as in Table 2.

#### False positives

An in-depth analysis of the false positives revealed that approximately 25% (131/543) had an EF between 41% and 49% on echocardiography, and hence closer to the designated threshold. Furthermore, 342 (63%) had a history or presence of at least one of the following: (permanent) atrial fibrillation, left bundle branch block, a pacemaker, and/or wide QRS complex (>120 ms). The rates of conduction/rhythm abnormalities for both false positives and true negatives are shown in Table 5.

**Table 5:** Rates of Comorbidities in the True Negative Vs False Positive Cohorts

	True Negatives (n = 1,963)	False Positives (n = 543)	*P* Value^a^
EF: 41%-49%	133 (6.8%)	131 (25%)	<0.001
Permanent AF	26 (1.3%)	26 (4.8%)	<0.001
AF	126 (6.4%)	108 (19.9%)	<0.001
LBBB	29 (1.5%)	74 (13.6%)	<0.001
Pacemaker	72 (3.7%)	103 (19.0%)	<0.001
Wide QRS complex^b^ (>120 ms)	247 (12.6%)	266 (49.0%)	<0.001

Values are n (%).

AF = atrial fibrillation; LBBB = left bundle branch block; other abbreviation as in Table 1.

a Chi-squared test.

b Either in addition to LBBB and pacemaker or isolated.

## Discussion

With this study, we aimed to prospectively validate the real-world potential of a novel digital stethoscope coupled with real-time AI analytics to identify patients with reduced EF (≤40%). Using single-lead ECG and PCG inputs collected from the stethoscope during a single 15-second recording, we demonstrated an AUROC of 0.85. This external, real-world validation of this technology supports its potential value as a noninvasive, scalable, and relatively low-cost method for identifying those at increased risk of reduced EF. Earlier diagnosis of a previously unrecognized low EF would allow for the earlier initiation of therapies proven to improve quality of life, decrease hospitalizations, and prolong survival.^22^

In our study, 210 (7.1%) of patients were found to have an EF ≤40%, consistent with prevalence rates from previous studies involving higher risk populations.^23^, ^24^, ^25^ The real-world implementation of the technology in this population demonstrated discriminatory capabilities comparable to other commonly utilized screening tests, such as mammography for breast cancer and cervical cytology for cervical cancer.^1^ An “augmented” logistic regression model including age, sex, presence of a pacemaker, and body mass index, in addition to low EF AI output, increased the AUROC from 0.85 to 0.87.

Arguably, there are several potential EF cutoff thresholds that could be of value when screening a population, with other solutions selecting EF of 35% and still others EF of 50%.^1^^,^^26^ We selected EF ≤40% after input from clinical experts and regulatory agencies. Also, this matches the American Heart Association/American College of Cardiology definition of heart failure with reduced EF, making it immediately actionable with strong evidence for the benefit of multiple guideline-directed medical therapies.^27^ The operating point of the US Food and Drug Administration-cleared low EF prediction model studied here was selected to optimize the sensitivity and specificity. When implemented in the setting of the current study, sensitivity and specificity were satisfactory, at ∼78% for both. With the low prevalence of individuals having an EF ≤40%, this resulted in a PPV of only ∼20%. Of note, one in 4 patients with a false positive low EF alert was found to have mildly reduced EFs between 40% and 50%. The NPV of the model was excellent at 98.0%. As more knowledge is gained through widespread implementation, consideration of the risk characteristics of the population being screened and the goals of screening will play a critical role in optimizing the technology further.

There is still a great deal to learn from the situation where the model predicts a low EF but echocardiography finds an EF of >40%. Beyond the fact that many have an EF between 40 and 50%, longitudinal follow-up in some studies of false positive patients has found a 4-fold increased risk of developing a low EF over the ensuing years relative to those with a true negative finding.^3^ In addition, it will be important to more thoroughly explore other clinical features that influence the performance of the model, such as underlying atrial fibrillation, presence of a pacemaker, or left bundle branch block, as identified in this study, in order to better optimize its performance.

Additionally, the usage of PCG for the detection of low EF in this study is new. The PCG model that is part of the final ensemble used for this study, by itself, is less predictive for low EF compared to the ECG model alone. However, PCG models based on new neural network architectures have been shown to have performance closer to ECG models.^12^ Understanding what features of the PCG and/or ECG are triggering predictions for low EF is the subject of future work. To make this possible, new machine learning (ML) approaches and analysis methods are required.

Currently, approximately 50 cardiovascular AI/ML medical devices have received US Food and Drug Administration clearance.^28^ Despite the increased focus of the scientific research community on health care-related AI research, there is a paucity of data available from prospective validation studies for US Food and Drug Administration-cleared AI/ML cardiovascular devices.^29^ Previous algorithms have shown promising performance in detecting LVSD, with area under the curve values ranging from 0.83 to 0.97. The majority of these studies were based on 12-lead ECGs obtained as part of routine clinical care.^1^^,^^30^ More recent studies have explored reduced-lead ECG (ie, single-lead) approaches. For example, Khunte et al^31^ reported a novel strategy adapting AI for noisy single-lead ECGs obtained from wearable and portable devices, achieving an AUROC of 0.87 for the detection of EF <40% on noise-augmented ECGs. Another study demonstrated the feasibility of detecting EF ≤40% using single-lead ECGs from smartwatches, achieving an AUROC of 0.89.^32^ Additionally, Bachtiger et al^14^ validated the use of AI applied to single-lead ECGs recorded with an ECG-enabled digital stethoscope, an earlier version of the device used in this study, achieving an AUROC of 0.85 in a multicenter real-world clinical setting. There is further promise for the application of these technologies in unique populations, such as those with peripartum cardiomyopathy (PPCM). A study conducted in Nigeria, which has the highest reported incidence of PPCM worldwide, demonstrated that AI-guided screening with a digital stethoscope significantly improved the diagnosis of pregnancy-related cardiomyopathy. In that study, the AI paired with a digital stethoscope had an AUROC of 0.98 for detecting EF <50% and 0.99 for detecting EF <40%, doubling the number of diagnosed cases compared to usual care.^33^^,^^34^ Interestingly, a significant increase in PPCM diagnosis was only observed for the AI-enabled single-lead ECG digital stethoscope model and not the 12-lead-based algorithm that was simultaneously studied.^34^ This was perhaps because the selected cutoff for determination of the primary outcome, where LVSD was defined as LVEF <50%, was not in line with the categorization used during the 12-lead model derivation (trained to detect an LVEF ≤35%).^1^ The potential for AI-guided screening using digital stethoscopes in lower-income countries, where access to advanced imaging modalities is limited, highlights the broader applicability and impact of this technology for early detection and management of cardiac conditions.^35^ Recent work has suggested that combining this tool with AI-guided echocardiographic imaging can lead to screening solutions at scale.^35^

### Study Limitations

Despite the promising results of this study, there are several limitations that need to be acknowledged. Firstly, while the discriminatory power, sensitivity, and specificity of the algorithm were good, there is substantial room for improvement. We believe that the addition of multiple ECG leads and/or the addition of clinical characteristics or other biomarkers such as BNP and NTproBNP could further optimize the identification of individuals with an undiagnosed low EF by potentially both improving sensitivity and PPV. Another limitation, shared by almost all ML models, is that the exact features of the ECG and PCG leading to the prediction of a low versus normal EF are not readily identifiable. In addition, the accuracy of the ground truth EF data from echocardiography and potential biases therein can influence the performance and reliability of the algorithm, although the influence of any variability in EF determination would likely only impact those with EFs close to (eg, ±5%) 40%. Furthermore, as the study population was comprised of patients already referred for an echocardiogram, the generalizability of our findings to the broader population may be limited. Finally, longitudinal studies examining patient outcomes are required to assess the clinical utility and cost-effectiveness of the diagnostic tool. Such studies would provide valuable insights into the long-term effectiveness and impact on patient management strategies.

## Conclusions

In conclusion, our study demonstrates the potential of a CNN model using single-lead ECG and PCG inputs collected with a digital stethoscope as a noninvasive tool for the detection of EF ≤40%. The algorithm showed good performance, with sensitivity and specificity comparable to other common medical screening tests. While our findings highlight the promise of this technology for improving detection rates, further research is needed to evaluate its impact on clinical outcomes and cost-effectiveness. Future work will focus on adapting the model for 3-lead input and optimizing algorithm performance using transformer models.

Perspectives **COMPETENCY IN PATIENT CARE AND PROCEDURAL SKILLS:** The CNN model using a digital stethoscope enables earlier detection of patients with reduced EF (EF ≤ 40%), facilitating timely intervention and management of left ventricular systolic dysfunction. This technology provides a noninvasive and accessible method for screening, which can be easily integrated into various clinical settings, including primary care and remote healthcare environments. The ability to identify at-risk patients early has the potential to reduce downstream healthcare costs associated with late-stage heart failure treatment.

**TRANSLATIONAL OUTLOOK:** For successful clinical translation, several factors need to be addressed. Further research is necessary to streamline the integration of this CNN model into existing clinical workflows, ensuring it complements current diagnostic procedures without causing disruptions. Additionally, validating the algorithm’s performance across diverse populations and different healthcare settings is crucial to ensure its generalizability and effectiveness. Addressing the clinical significance of false positives, particularly those with EF between 41% and 49%, will help refine the algorithm and enhance its predictive accuracy. Providing adequate training for healthcare providers to use this technology effectively will be essential for widespread adoption. Lastly, conducting longitudinal studies to assess the impact of early detection using this CNN model on long-term patient outcomes, including morbidity and mortality rates, will be vital in demonstrating its overall clinical utility.

## Funding support and author disclosures

Authors Guo, Kieu, Mathew, Prince, Lastowski, McDonough, Currie, Maidens, and Venkatraman are affiliated with Eko Health, Inc that develops digital stethoscopes, software, and algorithms to detect cardiovascular and pulmonary disease; hold company equity; and were responsible for algorithm development, collated data analysis, and codrafting of the manuscript. All other authors have reported that they have no relationships relevant to the contents of this paper to disclose.
