# Supplementary material for: Automated Detection of Reduced Ejection Fraction Using an ECG-Enabled Digital Stethoscope: A Large Cohort Validation
Source: JACC Adv. 2025 Feb 20;4(3):101619. doi: 10.1016/j.jacadv.2025.101619 (PMC11891699; doi:10.1016/j.jacadv.2025.101619)
Supplement: Supplemental Table 1 [file mmc1.docx]

**Supplemental Table 1.** Data contribution by participating site. No differences in performance across sites was observed

| **Site** | **N Subjects** | **Data Collection Period** |
| --- | --- | --- |
| Jefferson Einstein Philadelphia Hospital | 901 | 10/1/2020 - 4/29/2022 |
| Prairie Cardiovascular Consultants | 1766 | 6/22/21 - 5/24/22 |
| MedStar Health Research Institute | 97 | 4/6/2021 - 4/29/2022 |
| Ochsner Heart and Vascular Institute | 196 | 10/6/21 - 4/29/2022 |

**Supplemental Table 2.** Counts of recordings in the test dataset that had poor quality ECG and good quality ECG.

| **ECG/PCG Signal Quality** | | **Counts** | **Used for Analysis** |
| --- | --- | --- | --- |
| Poor Quality ECG | | 276 | No |
| Good Quality ECG | Good Quality PCG | 2333 | Yes |
|  | Poor Quality PCG | 351 | Yes |
| Total N | | 2960 |  |

The MI-CLAIM checklist

From: https://www.ncbi.nlm.nih.gov/pmc/articles/PMC7538196/

| **Before paper submission** |  | | |
| --- | --- | --- | --- |
| **Study design (Part 1)** | **Completed: page number** | | **Notes if not completed** |
| The clinical problem in which the model will be employed is clearly detailed in the paper. | Yes, page 5 | |  |
| The research question is clearly stated. | Yes, page 6 | |  |
| The characteristics of the cohorts (training and test sets) are detailed in the text. | Yes, page 10 and Table 1 | |  |
| The cohorts (training and test sets) are shown to be representative of real-world clinical settings. | Yes, pages 10 and 12 | |  |
| The state-of-the-art solution used as a baseline for comparison has been identified and detailed. | Yes, pages 13 and 14 | |  |
| **Data and optimization (Parts 2, 3)** | **Completed: page number** | | **Notes if not completed** |
| The origin of the data is described and the original format is detailed in the paper. | Y, pages 6 - 8 | |  |
| Transformations of the data before it is applied to the proposed model are described. | N | | To be included in separate manuscript detailing model development |
| The independence between training and test sets has been proven in the paper. | N | |  |
| Details on the models that were evaluated and the code developed to select the best model are provided. | N | |  |
| Is the input data type structured or unstructured? | **X Structured** | ☐ Unstructured |  |
| **Model performance (Part 4)** | **Completed: page number** | | **Notes if not completed** |
| The primary metric selected to evaluate algorithm performance (e.g., AUC, F-score, etc.), including the justification for selection, has been clearly stated. | Yes, page 8 | |  |
| The primary metric selected to evaluate the clinical utility of the model (e.g., PPV, NNT, etc.), including the justification for selection, has been clearly stated. | Y, page 8 | |  |
| The performance comparison between baseline and proposed model is presented with the appropriate statistical significance. | N/A | |  |
| **Model examination (Part 5)** | **Completed: page number** | | **Notes if not completed** |
| Examination technique 1[a](https://www.ncbi.nlm.nih.gov/pmc/articles/PMC7538196/table/T1/?report=objectonly#TFN2) | N/A |  | To be included in separate manuscript detailing model development |
| Examination technique 2[a](https://www.ncbi.nlm.nih.gov/pmc/articles/PMC7538196/table/T1/?report=objectonly#TFN2) | N/A |  |  |
| A discussion of the relevance of the examination results with respect to model/algorithm performance is presented. | N/A |  |  |
| A discussion of the feasibility and significance of model interpretability at the case level if examination methods are uninterpretable is presented. | N/A |  |  |
| A discussion of the reliability and robustness of the model as the underlying data distribution shifts is included. | N/A |  |  |
| **Reproducibility (Part 6): choose appropriate tier of transparency** |  |  | **Notes** |
| Tier 1: complete sharing of the code |  | ☐ |  |
| Tier 2: allow a third party to evaluate the code for accuracy/fairness; share the results of this evaluation | | ☐ |  |
| Tier 3: release of a virtual machine (binary) for running the code on new data without sharing its details | | ☐ |  |
| **Tier 4: no sharing** |  | **N/A** | Details of code sharing to be included in separate manuscript detailing model development |
